# Flow Cytometry Profiling of Plasmacytoid Dendritic Cell Neoplasms

**DOI:** 10.3390/cancers16112118

**Published:** 2024-06-01

**Authors:** Siba El Hussein, Wei Wang

**Affiliations:** 1 Department of Pathology, University of Vermont Larner College of Medicine, 111 Colchester Avenue, Burlington, VT 05401, USA; 2 Department of Hematopathology, University of Texas MD Anderson Cancer Center, 1515 Holcombe Blvd, Houston, TX 77030, USA

**Keywords:** flow cytometry analysis, plasmacytoid dendritic cells, blastic plasmacytoid dendritic cell neoplasm, BPDCN, mature plasmacytoid dendritic cell proliferation, MPDCP, AML with plasmacytoid dendritic cell differentiation, pDC-AML

## Abstract

**Simple Summary:**

There are three main types of neoplastic plasmacytoid dendritic cell (pDC) proliferations: blastic plasmacytoid dendritic cell neoplasm (BPDCN), acute myeloid leukemia with pDC differentiation (pDC-AML), and mature pDC proliferation (MPDCP). In this review, we focus on flow cytometry immunophenotyping analysis, discussing the immunophenotypes of each type of pDC proliferation, their differential diagnoses, and the challenges and pitfalls in evaluating these pDC proliferations.

**Abstract:**

In this review, we aim to provide a summary of the diverse immunophenotypic presentations of distinct entities associated with plasmacytoid dendritic cell (pDC) proliferation. These entities include the following: (1) blastic plasmacytoid dendritic cell neoplasm (BPDCN); (2) mature pDC proliferation (MPDCP), most commonly seen in chronic myelomonocytic leukemia (CMML); and (3) myeloid neoplasms with pDC differentiation, in which pDCs show a spectrum of maturation from early immature pDCs to mature forms, most commonly seen in acute myeloid leukemia (pDC-AML). Our aim is to provide a flow cytometry diagnostic approach to these distinct and sometimes challenging entities and to clarify the immunophenotypic spectrum of neoplastic pDCs in different disease presentations. In this review, we also cover the strategies in the evaluation of residual disease, as well as the challenges and pitfalls we face in the setting of immune and targeted therapy. The differential diagnosis will also be discussed, as blasts in some AML cases can have a pDC-like immunophenotype, mimicking pDCs.

## 1. Introduction

Plasmacytoid dendritic cells (pDCs) originate and mature in the bone marrow before being released into the systemic circulation and tissue, where they, although constituting less than 1% of total nucleated cells [1,2,3], act as an important player in the immune system [4].

Historically, the study of neoplastic pDC proliferation focused on “blastic plasmacytoid dendritic cell neoplasm” (BPDCN). However, recent studies have broadened our understanding of neoplastic pDC proliferation in association with myeloid neoplasms outside the context of BPDCN, that is, AML with pDC differentiation (pDC-AML). In addition, mature plasmacytoid dendritic cell proliferations (MPDCP) are also present, typically seen in the setting of chronic myelomonocytic leukemia (CMML). These distinct neoplastic pDC populations, along with neoplastic cells in BPDCN, incorporate immunophenotypic features from different stages of pDC maturation.

Flow cytometry plays an important role in the evaluation of pDCs. Bone marrow aspirates are the most common types of specimens used for flow cytometry evaluation of pDCs. Nevertheless, flow cytometry can also used in any other fresh specimen, including but not restricted to blood, lymph nodes, skin, etc. Flow cytometry analysis is a very sensitive and convenient method to detect normal and abnormal pDCs. It can also detect different maturation stages of pDCs based on characteristic patterns of antigen expression, which are typically not clearly appreciated by morphology and immunohistochemical stains. Flow cytometry analysis is particularly helpful in the assessment of minimal residual disease, which has proven to be of high clinical significance for disease risk stratification prior to and following stem cell transplantation [5]. Furthermore, different types of neoplastic pDC proliferations can be distinguished from each other using flow cytometry analysis. Finally, flow cytometry can also distinguish neoplastic pDCs from non-pDC neoplasms with a pDC-like immunophenotype.

In this article, we aim to provide the readership with an in-depth immunophenotypic profiling of the maturation pattern of physiologic pDCs and a detailed discussion of the immunophenotypic features of three broad classes of neoplastic pDC proliferations: (1) BPDCN and strategies in minimal residual disease analysis; (2) MPDCP; and (3) pDC-AML. AML with a pDC-like immunophenotype will also be discussed for the differential diagnosis.

## 2. The Suggested Flow Cytometry Panel and Gating Strategy for Evaluation of pDCs

**Figure 1:**
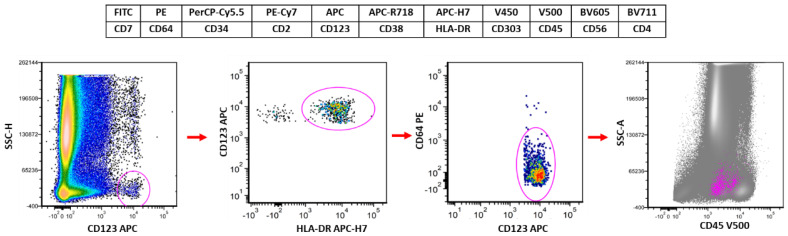
Flow cytometry panel and the gating strategy for evaluation of pDCs. Markers and their corresponding fluorochromes are listed. The gating strategy starts with CD123/SSC, followed by CD123/HLA-DR and CD123/CD64.

Before going into details about the immunophentype of pDCs, we briefly introduce the flow cytometry panel used in our daily practice and its common gating strategy (Figure 1). CD34 is included in the panel to highlight the earliest immature pDCs. CD123 and HLA-DR are included mainly for gating pDCs. CD64 is used to exclude monocytes, which can have bright CD123 and HLA-DR. CD303 is a specific pDC maker. CD4 is another pDC marker, although less specific than CD303. Other markers such as CD2, CD7, CD38 and CD56 are included mainly for distinguishing between reactive and neoplastic pDCs.

To analyze pDCs, CD123 bright cells are gated first, followed by a CD123/HLA-DR plot to exclude basophils (CD123+HLA-DR-). CD64 is then used to exclude monocytes, as some monocytes, especially non-classic monocytes, can have bright CD123. Finally, pDCs can be reflected on a CD45/SSC plot to show their distribution.

## 3. Normal and Neoplastic pDCs: Stages of Maturation

**Figure 2:**
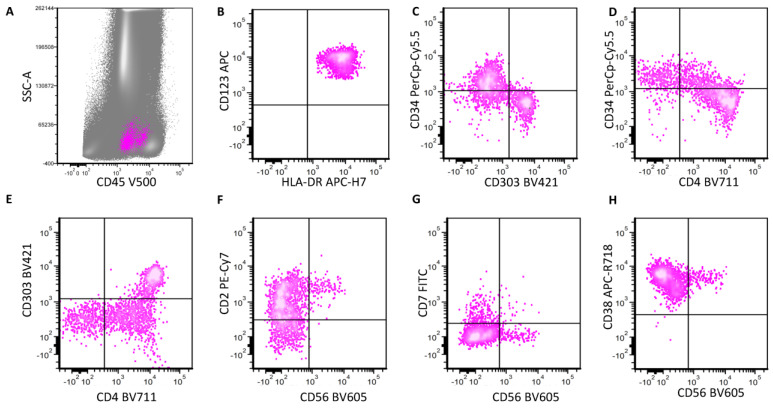
Normal pDC maturation in the bone marrow. pDCs are highlighted in pink. Early pDCs are in the traditional blast gate with dim CD45 expression on the CD45/SSC plot. As they mature, CD45 expression increases (**A**). CD123 and HLA-DR are brightly positive in all stages of maturation (**B**). Early pDCs are positive for CD34 and negative for CD303 and CD4 expression. As they mature, they become negative for CD34 expression and positive for CD4 and CD303 expression (**C**–**E**). Most pDCs in all maturation stages are positive for CD2 expression (**F**), including the CD56+ subset. A small subset of pDCs expresses CD7, and this subset is largely negative for CD56 expression (**G**). The CD56+ subset expresses bright CD38 (**H**).

pDCs are belived to originate from a common bone marrow hematopoietic progenitor, the same progenitor from which monocytes and myeloid dendritic cells derive [6]. Physiologic pDCs undergo three different stages of maturation: early, intermediate and late, each with a different expression pattern of immature markers (CD34 and CD117) and pDC-specific markers such as CD303 (BDCA-2) and CD304 [7,8,9,10] (Figure 2). The maturation stages and the expression pattern of key markers in pDCs are illustrated in our recent review [8]. Briefly, the earliest pDCs express CD34 and CD117, with no CD4, CD303 and CD304 expression [8]. CD56 is also negative in the early stages. As cells mature, pDCs lose CD34 and CD117 expression; simultaneously, they gain CD4, CD303 and CD304 expression at the intermediate stage [8]. During the late stages of maturation, pDCs show the highest expression of CD4, CD303 and CD304 [8]. CD38 is bright at early stages, and its expression gradually decreases but stays positive as cells mature [8]. CD45 levels gradually increase as pDCs mature [8]. A small subset of pDCs at the intermediate stage expresses CD56, and CD56+ pDCs are positive for CD2 and CD5 expression [10].

CD123 expression is slightly dimmer in the earliest pDCs. HLA-DR is transiently decreased when cells mature from early to intermediate stage. Overall, pDCs show a relatively strong expression of CD123 and HLA-DR during all stages of maturation. Thus, these two markers are most commonly used to gate pDCs (both mature and immature) in flow cytometry analysis.

Neoplastic pDCs in different diseases are believed to derive from different stages of pDC maturation. BPDCN is considered to derive from the CD56+ pDC subset [11,12]. MPDCP associated with myeloid neoplasms, in which pDCs are fully mature, derives from the late stages of the pDC maturation spectrum [1,13,14]. For myeloid neoplasms with pDC differentiation, mainly seen in pDC-AML [9,15,16], but also in myelodysplastic neoplasm (MDS) [9,17] and myeloproliferative neoplasm (MPN) [16,17], pDCs show a spectrum of maturation ranging from early pDCs to fully mature pDCs and are believed to originate from early pDC precursors [8].

## 4. BPDCN

**Figure 3:**
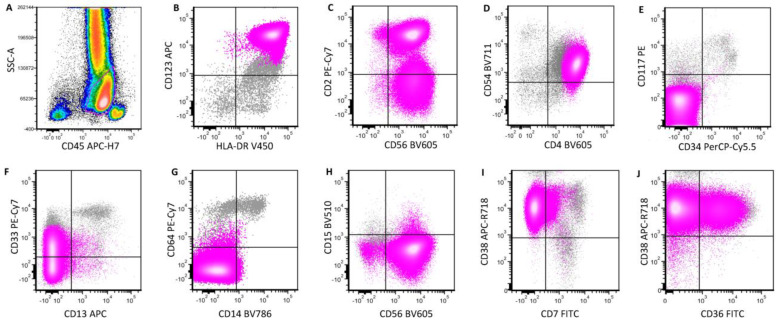
A representative case of BPDCN in bone marrow. Neoplastic pDCs (highlighted in pink) are positive for CD45 expression (**A**) and are characterized by a uniform expression of CD123, HLA-DR (**B**), CD56 (**C**) and CD4 (**D**). They are negative for CD34, CD117 (**E**), CD13 (**F**), CD14, CD64 (**G**) and CD15 (**H**) expression. CD33 (**F**), CD7 (**I**) and CD36 (**J**) are positive in a subset of cells, respectively.

BPDCN is a hematological neoplasm consisting of aberrant pDC proliferation, characterized by a high frequency of cutaneous involvement and systemic dissemination. The neoplastic cells in BPDCN typically express CD4, CD56, CD123, CD304, HLA-DR, TCL1 and TCF4 [2]. CD303 is variably expressed. CD56 expression is a characteristic feature of BPDCN although it may be negative in very rare cases, particularly in pediatric BPDCN [2,18]. A subset of cases shows CD2 and CD7 expression, and a smaller subset shows CD5 expression [2]. Neoplastic cells in BPDCN are negative for lineage-specific antigens, including CD19 for B-cells, surface and cytoplasmic CD3 for T-cells, myeloperoxidase for myeloid cells, and CD64 for monocytic cells [18]. The neoplastic cells in the majority of BPDCN cases display a similar level of expression of CD45 as that of granulocytes, or slightly higher [2]. A small subset of cases may show lower expression of CD45, dimmer than that of granulocytes [2]. Of note, many markers frequently expressed in BPDCN, such as CD4, CD56, CD123, CD304 and HLA-DR, are not lineage-specific for pDCs. Although the expression of these markers raises the possibility of BPDCN, a definitive diagnosis relies on the demonstration of tumor cells expressing more specific pDC markers such as CD303, TCF4 and TCL1. Dual expression of TCF4 and CD123 by immunohistochemistry (IHC) is highly sensitive and specific for the pDC lineage. For laboratories that do not have these pDC-specific antibodies, extensive workup to rule out other lineages (myeloid, monocytic, T-cell) is required for the diagnosis of BPDCN. A representative case of BPDCN is illustrated in Figure 3.

The value of flow cytometry analysis, particularly in the setting of BPDCN minimally involving bone marrow or concurrently presenting with other myeloid neoplasms, has been previously highlighted [11]. In this scenario, the bone marrow involved by the myeloid neoplasm is typically hypercellular, and the BPDCN component may be challenging to identify based on the morphologic assessment of the core biopsy alone [12,19]. A pDC population in this setting is more likely to be detected by flow cytometry analysis, which would trigger further evaluation to determine the presence of a concurrent BPDCN along with the established hematologic neoplasm [12]. Thus, a flow cytometry panel with core pDC markers is recommended for the evaluation of myeloid neoplasms. As discussed above, two valuable and core markers that can recognize pDCs are CD123 and HLA-DR. Once a prominent population is highlighted by CD123 and HLA-DR, further markers can be added by flow cytometry analysis in this setting, including pDC-specific markers such as CD303, TCF4 and TCL1 by flow cytometry or IHC. Of note, TCL1 is expressed in most normal B cells; thus, B cells need to be ruled out before designating TCL1-positive cells as pDCs.

## 5. BPDCN Minimal Residual Disease

Normal bone marrows commonly have a small population of reactive pDCs. In the case of BPDCN after therapy for evaluation of minimal residual disease, the distinction between reactive pDCs and residual BPDCN can be challenging. Similar to their neoplastic counterpart, reactive pDC are positive for CD4, CD123, CD303, HLA-DR, TCL1 and TCF4 expression, and they also lack expression of lineage-specific antigens [8].

**Figure 4:**
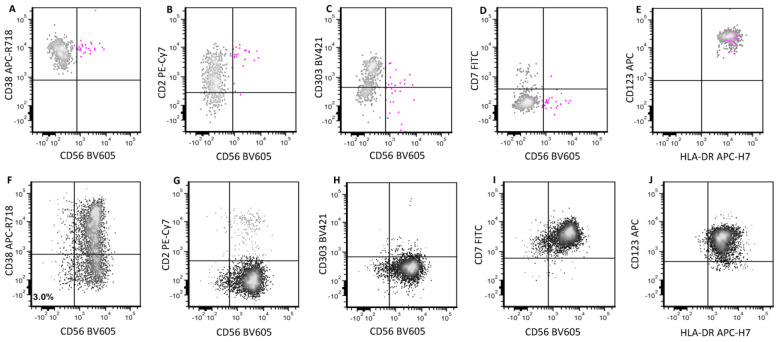
The immunophenotypic difference between CD56+ reactive and neoplastic pDCs. Bone marrow aspirates are analyzed. The upper panel shows reactive CD56+ pDCs (pink) that are brightly positive for CD38 (**A**) and CD2 (**B**). They are partially positive for CD303 (**C**). CD7 is negative (**D**). CD123 and HLA-DR are brightly positive (**E**). The lower panel shows neoplastic CD56+ pDCs (black) in post-treatment evaluation for minimal residual disease in a case of BPDCN. Cells show uniform CD56 with decreased CD38 expression (**F**). In contrast to reactive pDCs, they are negative for CD2 (**G**) and positive for CD7 expression (**I**). CD303 is completely negative (**H**). CD123 level (**J**) is decreased after treatment.

Most BPDCN cases express CD56, which is the marker widely used to distinguish neoplastic from reactive pDCs. However, CD56 expression is not confined to neoplastic pDCs and is present in a small subset of normal/reactive pDCs [2,10,20,21]. In a previous study, we explored the immunophenotypic difference between CD56+ reactive and neoplastic pDCs [2]. This CD56-positive subset of reactive pDCs shows positivity for CD2 and CD303 (often partial) expression with bright CD38 expression. They are negative for CD7 [2,10,20,21], an immunophenotypic pattern that is distinct from that of neoplastic BPDCN cells, which are more often negative for CD2 and positive for CD7. Decreased or negative CD38 and CD303 expression is common in BPDCN. Accordingly, implementing a flow cytometry panel (Figure 1) composed of markers (CD2, CD7, CD38, CD303), that take advantage of these immunophenotypic differences, is helpful in reliably distinguishing BPDCN cells from reactive pDCs [2]. A representative case of MRD evaluation is illustrated in Figure 4.

**Figure 5:**
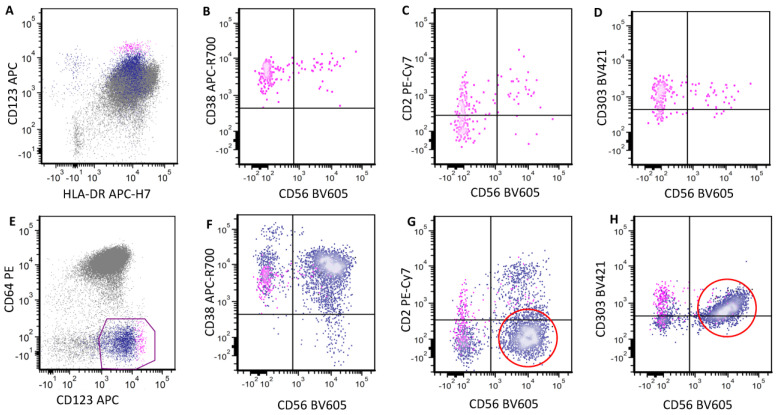
Neoplastic pDCs show decreased CD123 expression after targeted therapy. This is a post-treatment specimen from a BPDCN patient who was treated with IMGN632, a CD123-targeting antibody–drug conjugate. When only CD123 bright cells were gated (pink, (**A**)) in this bone marrow, they were 0.1% of cells. The majority of these cells are negative for CD56 (**B**), positive for CD2 (**C**) and CD303 (**D**), consistent with reactive pDCs. However, when both CD123+ moderate and bright cells are gated (1.2% of total cells, blue and pink, (**E**)), many are CD56-positive (**F**) with negativity for CD2 expression (**G**). CD303 is positive (**H**). The overall findings are consistent with aberrant pDCs and residual bPDCN.

CD123 is the marker typically used to gate and highlight pDCs for further evaluation by flow cytometry, given its bright and uniform expression in pDCs. One caveat associated with post-treatment MRD evaluation is that CD123 levels can be significantly decreased after CD123-targeted therapy. CD123-targeted therapy, whether antibody-based or CART-based, is widely used in the treatment of BPDCN patients [22]. In our experience, CD123 still maintains its high level of expression in residual tumor cells after targeted therapy, but occasionally we have encountered cases with significantly decreased CD123 expression. In such cases, the CD123 gate should be expanded to include CD123 dimmer cells. Alternatively, CD56+CD64-HLA-DR+ can be used for gating, as most BPDCN cases are positive for CD56. A representative case is illustrated in Figure 5.

## 6. Mature Plasmacytoid Dendritic Cell Proliferation

Mature plasmacytoid dendritic cell proliferation (MPDCP) [18] in the bone marrow or extra-medullary sites, such as lymph nodes and skin [1,17,23,24,25], is composed of nodules of mature pDCs. Previous studies have suggested MPDCP to be clonally related to the concurrent myeloid neoplasm [1,23,25], most commonly CMML and occasionally MDS [26,27] or AML [3]. Myeloid neoplasms, particularly CMML, with pDC proliferations have been shown to have a higher risk of transformation to AML [1].

**Figure 6:**
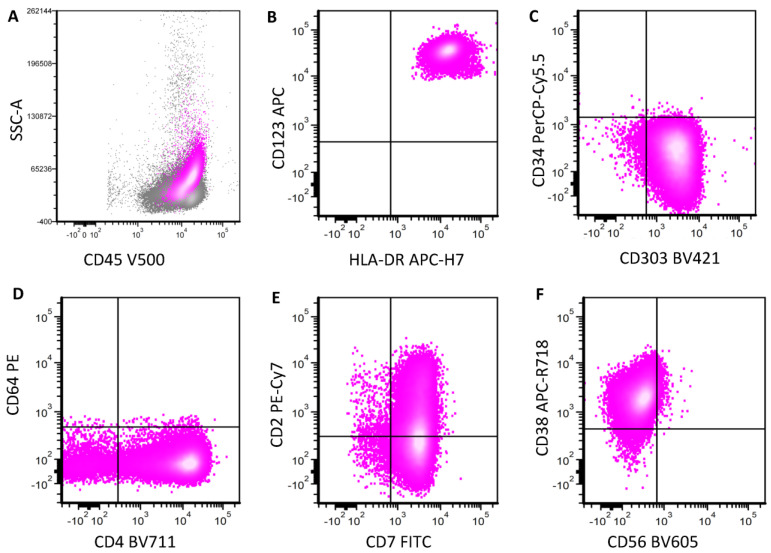
The immunophenotype of pDCs in mature pDC proliferations. This is a patient with a recent diagnosis of chronic myelomonocytic leukemia who presented with lymphadenopathy. A lymph node biopsy showed myeloid sarcoma with a pDC population ((**A**), pink), accounting for 50% of total cells. They are brightly positive for CD123 and HLA-DR (**B**). CD303 (**C**) and CD4 (**D**) are positive. They are negative for the immature marker CD34. CD56 (**F**) and CD64 (**D**) are negative. CD2 is partially positive. When compared to normal pDCs, they show an aberrant immunophenotype with increased CD7 (**E**) and decreased CD38 expression (**F**).

The immunophenotypic profile of MPDCP is similar to that of reactive mature pDCs in the late stages of maturation, characterized by the expression of CD123 (bright and homogeneous), CD303, CD304 and TCL1, and the absence of immature marker(CD34 and CD117) expression. TdT expression is negative [8]. Expression of T-cell markers (CD2, CD5, CD7) and myeloid markers (CD13, CD33) may be seen [14,17,28]. CD56 is typically negative, and if positive, it is only seen in a small subset with dim expression, different from the pattern seen in BPDCN with strong and homogeneous positivity [2,13,14]. A representative case is illustrated in Figure 6.

## 7. Myeloid Neoplasms with pDC Differentiation

Myeloid neoplasms may undergo pDC differentiation, most commonly seen in AML (pDC-AML) [29,30]. 

pDC-AML constitutes about 3–5% of all AMLs [29,30]. In pDC-AML, the median number of pDCs is 6.6% (range, 2–26.3%), according to one study [30]. Unlike MPDCP, in which pDCs are mature, pDCs in pDC-AML are characterized by a full spectrum of maturation composed of various stages of pDCs, including the early forms expressing CD34 and CD117 with low levels of CD4 and CD303 and the late/mature forms that are completely negative for CD34 and CD117 expression with high expression of CD4 and CD303 [8]. Genetically, pDCsand the myeloblastic component of pDC-AML show evidence of clonal relatedness, indicating that they derive from the same progenitors [29].

**Figure 7:**
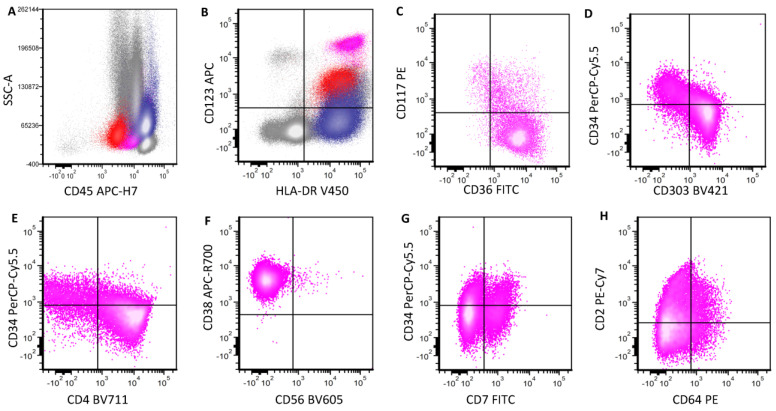
A representative case of pDC-AML. This is a case of acute myelomonocytic leukemia with pDC differentiation in the bone marrow. In (**A**), three populations are highlighted: red: myeloblasts; pink: pDCs; blue: monocytes. pDCs are brightly positive for CD123 and HLA-DR expression (**B**). They show a spectrum of maturation, with early immature cells positive for CD117 and CD34 while negative for CD303 and CD4 (**C**–**E**). Mature cells with an opposite immunophenotype are also present. pDCs are negative for CD56 (**F**). They show partial expression of CD7 (**G**), CD2 and CD64 (**H**).

pDC-AML is composed of two aberrant populations, myeloblasts and pDCs. Flow cytometry analysis is sensitive and convenient to detect and distinguish pDCs and myeloid precursors in pDC-AML. Myeloblasts in pDC-AML often show an immature myeloid or myelomonocytic immunophenotype with the expression of CD34, CD117, HLA-DR and TdT. CD123 expression is often increased. While the myeloid and pDC components share the expression of certain markers, such as CD34, CD117 and CD123, myeloid blasts do not express pDC-specific markers, such as CD303 [8]. CD123 expression in myeloblasts is increased, but its level is still lower than that seen in pDCs. For MRD evaluation in pDC-AML, focusing on immunophenotypic aberrancies in myeloblasts is recommended, as pDCs in pDC-AML often have an immunophenotype that cannot be distinguished from reactive pDCs. A representative case of pDC-AML is illustrated in Figure 7.

## 8. AML and T-ALL with a pDC-like Phenotype

**Figure 8:**
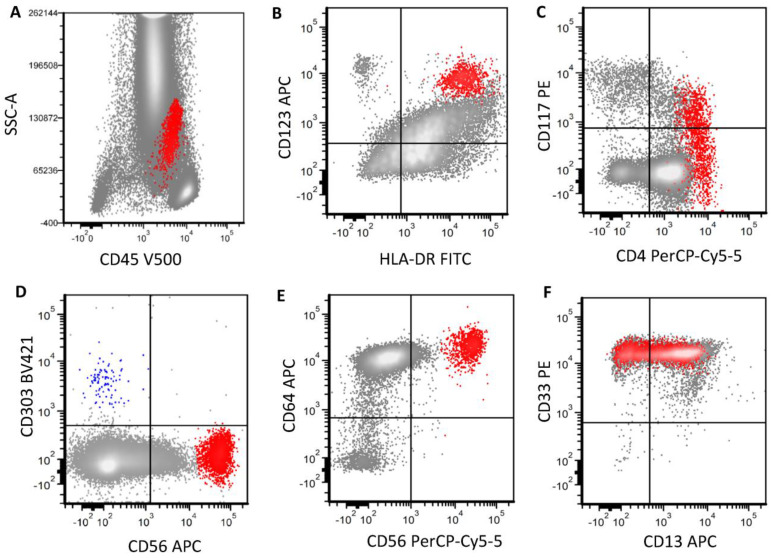
A representative case of AML with a pDC-like immunophenotype. Blasts ((**A**), red) in this bone marrow show strong CD123 and HLA-DR expression (**B**). CD4 (**C**) and CD56 (**D**) are strongly positive. This immunophenotypic feature mimics BPDCN. However, they are completely negative for CD303 expression (**D**), with bright CD64 expression (**E**), which is not consistent with pDCs. The bright CD64 coupled with bright CD33 (**F**) and partial CD13 expression are consistent monocytic cells. Blue population in D is reactive pDCs in the background with CD303 expression.

A subset of AML cases, especially those with monocytic features, may rarely exhibit a pDC-like immunophenotype with strong and homogeneous CD4, CD56 and CD123 expression, mimicking BPDCN [31,32,33]. Although cutaneous involvement is a characteristic feature of BPDCN, BPDCN with a leukemic presentation at onset without cutaneous involvement is an established phenomenon [34,35,36] that occurs in about 7% of BPDCN cases [13]. Thus, in the setting of an acute leukemia with a “pDC-like phenotype”, the main task is to determine whether it is a case of BPDCN or AML with a pDC-like immunophenotype. Distinctly from BPDCN, blasts in these cases are not bona fide pDCs, and they are negative for pDC-specific markers CD303 and TCF4. Additionally, they often express monocytic markers such as CD64 and/or CD14. A representative case is illustrated in Figure 8.

Additionally, a subset of T-ALLs lacks CD34 expression, and some T-ALLs can express CD56, overlapping with a subset of BPDCN that expresses T-cell markers (such as CD2, CD5, CD4, CD7 and TDT) [33]. Lineage-defining markers are critical for this differential diagnosis. Cytoplasmic CD3 is positive in T-ALLs and negative in BPDCN. On the other hand, CD303, TCF4 and TCL1 are highly specific to pDCs, and they are negative in T-ALLs.

## 9. Conclusions

pDC maturation can be divided into three physiologic stages. Likewise, three types of neoplastic pDC proliferations mirroring the phenotypic characteristics of each stage of pDC maturation exist: (1) pDC-AML (early stage of maturation); (2) BPDCN (intermediate stage of maturation); and (3) MPDCP (late stage of maturation). These three neoplastic pDC proliferations show distinct immunophenotypes and associated myeloid neoplasms. It is important to distinguish them, as their treatment and prognosis are different. Flow cytometry represents a powerful and convenient tool that helps resolve diagnostic challenges in this setting. pDCs in all these three diseases are positive for CD4, CD123 and HLA-DR. Different from pDCs in BPDCN and MPDCP, pDCs in pDC-AML show a spectrum of maturation. CD34, an immature marker that highlights early pDCs, is positive in pDC-AML and negative in BPDCN and MPDCP. CD56 is characteristically positive in BPDCN but usually negative in pDC-AML and MPDCP, although rare cases in the latter two diseases can have dim and/or partial CD56 expression. A significant subset of BPDCN cases show negative CD303, which is often positive in pDC-AML (subset) and MPDCP. TCL1 is frequently negative in pDC-AML and positive in BPDCN and MPDCP. Although molecular characteristics are not the scoop of this review, the mutation profiles are different in these three diseases. pDC-AML often shows the *RUNX1* mutation, whereas mutations in *TET2* and *ASCL1* are commonly seen in BPDCN and MPDCP.

## References

[1] Lucas N., Duchmann M., Rameau P., Noël F., Michea P., Saada V., Kosmider O., Pierron G., Fernandez-Zapico M.E., Howard M.T. (2019). Biology and prognostic impact of clonal plasmacytoid dendritic cells in chronic myelomonocytic leukemia. Leukemia.

[2] Wang W., Khoury J.D., Miranda R.N., Jorgensen J.L., Xu J., Loghavi S., Li S., Pemmaraju N., Nguyen T., Medeiros L.J. (2021). Immunophenotypic characterization of reactive and neoplastic plasmacytoid dendritic cells permits establishment of a 10-color flow cytometric panel for initial workup and residual disease evaluation of blastic plasmacytoid dendritic cell neoplasm. Haematologica.

[3] Xiao W., Goldberg A.D., Famulare C.A., Devlin S.M., Nguyen N.T., Sim S., Kabel C.C., Patel M.A., McGovern E.M., Patel A. (2019). Loss of plasmacytoid dendritic cell differentiation is highly predictive for post-induction measurable residual disease and inferior outcomes in acute myeloid leukemia. Haematologica.

[4] Reizis B. (2019). Plasmacytoid Dendritic Cells: Development, Regulation, and Function. Immunity.

[5] Murthy H.S., Zhang M.-J., Chen K., Ahmed S., Deotare U., Ganguly S., Kansagra A., Michelis F.V., Nishihori T., Patnaik M. (2023). Allogeneic hematopoietic cell transplantation for blastic plasmacytoid dendritic cell neoplasm: A CIBMTR analysis. Blood Adv..

[6] Collin M., Bigley V. (2018). Human dendritic cell subsets: An update. Immunology.

[7] Martin-Martin L., Almeida J., Hernandez-Campo P.M., Sanchez M.L., Lecrevisse Q., Orfao A. (2009). Immunophenotypical, morphologic, and functional characterization of maturation-associated plasmacytoid dendritic cell subsets in normal adult human bone marrow. Transfusion.

[8] El Hussein S., Wang W. (2023). Plasmacytoid dendritic cells in the setting of myeloid neoplasms: Diagnostic guide to challenging pathologic presentations. Br. J. Haematol..

[9] Huang Y., Wang Y., Chang Y., Yuan X., Hao L., Shi H., Lai Y., Huang X., Liu Y. (2020). Myeloid Neoplasms with Elevated Plasmacytoid Dendritic Cell Differentiation Reflect the Maturation Process of Dendritic Cells. Cytom. A.

[10] Comeau M.R., Van der Vuurst de Vries A.-R., Maliszewski C.R., Galibert L. (2002). CD123bright plasmacytoid predendritic cells: Progenitors undergoing cell fate conversion?. J. Immunol..

[11] Pemmaraju N., Kantarjian H.M., Khoury J.D., Loghavi S., O’Brien S., Cortes J.E., Garcia-Manero G., Jabbour E., Verstovsek S., Jain N. (2019). Blastic Plasmacytoid Dendritic Cell Neoplasm (BPDCN) Commonly Presents in the Setting of Prior or Concomitant Hematologic Malignancies (PCHM): Patient Characteristics and Outcomes in the Rapidly Evolving Modern Targeted Therapy Era. Blood.

[12] El Hussein S., Yabe M., Wang W., Pemmaraju N., Loghavi S., Jelloul F.Z., Fang H., Medeiros L.J., Burack W.R., Evans A.G. (2022). Blastic plasmacytoid dendritic cell neoplasm (BPDCN) arising in the setting of polycythemia vera (PV): An illustration of the emerging role of flow cytometry analysis in monitoring progression of myeloproliferative neoplasms. EJHaem.

[13] Facchetti F., Cigognetti M., Fisogni S., Rossi G., Lonardi S., Vermi W. (2016). Neoplasms derived from plasmacytoid dendritic cells. Mod. Pathol..

[14] Vitte F., Fabiani B., Benet C., Dalac S., Balme B., Delattre C., Vergier B., Beylot-Barry M., Vignon-Pennamen D., Ortonne N. (2012). Specific skin lesions in chronic myelomonocytic leukemia: A spectrum of myelomonocytic and dendritic cell proliferations: A study of 42 cases. Am. J. Surg. Pathol..

[15] Zalmaï L., Viailly P.-J., Biichle S., Cheok M., Soret L., Angelot-Delettre F., Petrella T., Collonge-Rame M.-A., Seilles E., Geffroy S. (2021). Plasmacytoid dendritic cells proliferation associated with acute myeloid leukemia: Phenotype profile and mutation landscape. Haematologica.

[16] Hamadeh F., Awadallah A., Meyerson H.J., Beck R.C. (2020). Flow Cytometry Identifies a Spectrum of Maturation in Myeloid Neoplasms Having Plasmacytoid Dendritic Cell Differentiation. Cytom. B Clin. Cytom..

[17] Vermi W., Facchetti F., Rosati S., Vergoni F., Rossi E., Festa S., Remotti D., Grigolato P., Massarelli G., Frizzera G. (2004). Nodal and extranodal tumor-forming accumulation of plasmacytoid monocytes/interferon-producing cells associated with myeloid disorders. Am. J. Surg. Pathol..

[18] Khoury J.D., Solary E., Abla O., Akkari Y., Alaggio R., Apperley J.F., Bejar R., Berti E., Busque L., Chan J.K.C. (2022). The 5th edition of the World Health Organization Classification of Haematolymphoid Tumours: Myeloid and Histiocytic/Dendritic Neoplasms. Leukemia.

[19] Khan A.M., Munir A., Raval M., Mehdi S. (2019). Blastic plasmacytoid dendritic cell neoplasm in the background of myeloproliferative disorder and chronic lymphocytic leukaemia. BMJ Case Rep..

[20] Osaki Y., Yokohama A., Saito A., Tahara K., Yanagisawa K., Ogawa Y., Ishizaki T., Mitsui T., Koiso H., Takizawa M. (2013). Characterization of CD56+ dendritic-like cells: A normal counterpart of blastic plasmacytoid dendritic cell neoplasm?. PLoS ONE.

[21] Petrella T., Comeau M.R., Maynadie M., Couillault G., De Muret A., Maliszewski C.R., Dalac S., Durlach A., Galibert L. (2002). ‘Agranular CD4+ CD56+ hematodermic neoplasm’ (blastic NK-cell lymphoma) originates from a population of CD56+ precursor cells related to plasmacytoid monocytes. Am. J. Surg. Pathol..

[22] Wilson N.R., Konopleva M., Khoury J.D., Pemmaraju N. (2021). Novel Therapeutic Approaches in Blastic Plasmacytoid Dendritic Cell Neoplasm (BPDCN): Era of Targeted Therapy. Clin. Lymphoma Myeloma Leuk..

[23] Machan S., Alonso-Dominguez J.M., Sanchez Garcia F.J., Nieves Salgado R., Soto C., Castro Y., Pajares R., Manso R., Santonja C., Serrano Del Castillo C. (2022). Plasmacytoid Dendritic Cell Dermatosis Associated to Myeloproliferative/Myelodysplastic Neoplasms. Am. J. Surg. Pathol..

[24] Dargent J.-L., Henne S., Pranger D., Balzarini P., Sartenaer D., Bulliard G., Rack K., Facchetti F. (2016). Tumor-forming plasmacytoid dendritic cells associated with myeloid neoplasms. Report of a peculiar case with histopathologic features masquerading as lupus erythematosus. J. Cutan. Pathol..

[25] Tzankov A., Hebeda K., Kremer M., Leguit R., Orazi A., van der Walt J., Gianelli U. (2017). Plasmacytoid dendritic cell proliferations and neoplasms involving the bone marrow: Summary of the workshop cases submitted to the 18th Meeting of the European Association for Haematopathology (EAHP) organized by the European Bone Marrow Working Group, Basel 2016. Ann. Hematol..

[26] Chan A., Liu Y., Devlin S., Gao Q., Baik J., Sigler A., Londono D., Arcila M., Levine R., Dogan A. (2022). Reduced Plasmacytoid Dendritic Cell Output Is Associated With High Risk in Low-grade Myelodysplastic Syndrome. Hemasphere.

[27] Ma L., Delforge M., van Duppen V., Verhoef G., Emanuel B., Boogaerts M., Hagemeijer A., Vandenberghe P. (2004). Circulating myeloid and lymphoid precursor dendritic cells are clonally involved in myelodysplastic syndromes. Leukemia.

[28] Marafioti T., Paterson J.C., Ballabio E., Reichard K.K., Tedoldi S., Hollowood K., Dictor M., Hansmann M.-L., Pileri S.A., Dyer M.J. (2008). Novel markers of normal and neoplastic human plasmacytoid dendritic cells. Blood.

[29] Xiao W., Chan A., Waarts M.R., Mishra T., Liu Y., Cai S.F., Yao J., Gao Q., Bowman R.L., Koche R.P. (2021). Plasmacytoid dendritic cell expansion defines a distinct subset of RUNX1-mutated acute myeloid leukemia. Blood.

[30] Wang W., Xu J., Khoury J.D., Pemmaraju N., Fang H., Miranda R.N., Yin C.C., Hussein S.E., Jia F., Tang Z. (2022). Immunophenotypic and Molecular Features of Acute Myeloid Leukemia with Plasmacytoid Dendritic Cell Differentiation Are Distinct from Blastic Plasmacytoid Dendritic Cell Neoplasm. Cancers.

[31] Wang W., Thakral B. (2020). CD123+CD4+CD56+ neoplasm: Blastic plasmacytoid dendritic cell neoplasm or acute myeloid leukemia?. Blood.

[32] Germans S.K., Chen W. (2024). The great mimicker: Leukemic presentation of blastic plasmacytoid dendritic cell neoplasm with PVT1::SUPT3H fusion. EJHaem.

[33] Cuglievan B., Connors J., He J., Khazal S., Yedururi S., Dai J., Garces S., Quesada A.E., Roth M., Garcia M. (2023). Blastic plasmacytoid dendritic cell neoplasm: A comprehensive review in pediatrics, adolescents, and young adults (AYA) and an update of novel therapies. Leukemia.

[34] Pagano L., Valentini C.G., Pulsoni A., Fisogni S., Carluccio P., Mannelli F., Lunghi M., Pica G., Onida F., Cattaneo C. (2013). Blastic plasmacytoid dendritic cell neoplasm with leukemic presentation: An Italian multicenter study. Haematologica.

[35] Wang H., Cao J., Hong X. (2012). Blastic plasmacytoid dendritic cell neoplasm without cutaneous lesion at presentation: Case report and literature review. Acta Haematol..

[36] Rauh M.J., Rahman F., Good D., Silverman J., Brennan M.K., Dimov N., Liesveld J., Ryan D.H., Burack W.R., Bennett J.M. (2012). Blastic plasmacytoid dendritic cell neoplasm with leukemic presentation, lacking cutaneous involvement: Case series and literature review. Leuk. Res..

